# Transition and factors associated with the level of physical activity combined with sedentary behavior of the elderly: A longitudinal study

**DOI:** 10.7705/biomedica.5108

**Published:** 2020-06-30

**Authors:** Lilane Maria Alves Silva, Darlene Mara dos Santos Tavares, Leiner Resende Rodrigues

**Affiliations:** 1 Federal University of Triângulo Mineiro, Postgraduate Program in Health Care, Uberaba, Minas Gerais, Brazil Federal University of Triângulo Mineiro Postgraduate Program in Health Care UberabaMinas Gerais Brazil

**Keywords:** Motor activity, sedentary behavior, health of the elderly, risk factors, longitudinal studies, health surveys, actividad motora, conducta sedentaria, salud del anciano, factores de riesgo, estudios longitudinales, encuestas epidemiológicas.

## Abstract

**Introduction:**

Physical activity and sedentary behavior are emerging issues in public health, especially in developing countries.

**Objective:**

To verify transition and factors related to physical activity combined with sedentary behavior among the elderly followed for 24 months.

**Materials and methods:**

We conducted a longitudinal observational study with people aged 60 years or over living in the urban area of Uberaba, Brazil. We collected the data from sociodemographic, health, and physical tests in 2014 and 2016 using the Mini-Mental State Examination (MMSE), the Katz Index, the Lawton and Brody Scale, the Short Physical Performance Battery (SPPB), and the International Physical Activity Questionnaire (IPAQ). For the combined evaluation we considered a cutoff point of 150 minutes of physical activity per week and the percentile 75 (420 minutes/day) for sedentary behavior constituting the groups: Unsatisfactory (insufficient sum of physical activity and sedentary behavior), intermediate (loss of only one of the two components) and satisfactory (sufficient sum of physical activity and sedentary behavior). The statistical descriptive and inferential analysis was performed using the Statistical Package for Social Sciences^™^, version 21.0, considering p<0.05.

**Results:**

Of the 374 elderly, 61 (16.3%) improved their physical activity and sedentary behavior condition, 226 (60.4%) remained in the same category and 87 (23.3%) got worse. Unsatisfactory levels of physical activity and sedentary behavior were related to the eldest group (p=0.031), the absence of professional activity (p<0.001), the dependence for instrumental activities of daily living (p=0.013), and a worse physical performance (p<0.001).

**Conclusion:**

Our results showed a relationship between sociodemographic and health factors with physical activity and sedentary behavior, reiterating the need for further research on the subject.

## Resumen

**Introducción.:**

La actividad física y el comportamiento sedentario son temas emergentes de salud pública, especialmente en los países en desarrollo.

**Objetivo.:**

Verificar la transición y los factores relacionados con la actividad física y el comportamiento sedentario entre ancianos durante un seguimiento de 24 meses.

**Materiales y métodos.:**

Se llevó a cabo un estudio longitudinal y observacional en ancianos de 60 años o más residentes en Uberaba, Minas Gerais, Brasil. Los datos sociodemográficos, de salud y de los exámenes físicos se recolectaron en el 2014 y el 2016 utilizando el *Mini-Mental State Examination* (MMSE), el índice de Katz, la escala de Lawton y Brody, la *Short Physical Performance Battery* (SPPB) y el *International Physical Activity Questionnaire,* (IPAQ). Para la evaluación combinada se consideró el punto de corte de 150 minutos semanales de actividad física y el percentil 75 (420 minutos/día) para el comportamiento sedentario, lo que arrojó los siguientes grupos: insatisfactorio (suma insuficiente de actividad física y comportamiento sedentario), intermedio (pérdida en uno solo de los dos componentes) y satisfactoria (suma suficiente de actividad física y comportamiento sedentario). El análisis estadístico descriptivo e inferencial se hizo con el *Statistical Package for Social Sciences* ^™^, versión 21.0, p<0,05.

**Resultados.:**

De los 374 ancianos, 61 (16,3 %) mejoraron su condición de actividad física y comportamiento sedentario, 226 (60,4 %) permanecieron en la misma categoría y 87 (23,3 %) empeoraron. Los niveles insatisfactorios de actividad física y comportamiento sedentario se asociaron con una mayor edad (p=0,031), la ausencia de actividad profesional (p<0,001), la dependencia para las actividades instrumentales de la vida diaria (p=0,013) y un peor rendimiento físico (p<0,001).

**Conclusiones.:**

Los resultados evidenciaron una relación entre los factores sociodemográficos y de salud con la actividad física y el comportamiento sedentario, lo que reitera la necesidad de nuevos estudios sobre la salud del anciano.

Population aging is a manifestation of world order. In Brazil, this process is ostensibly taking place at a rapid pace. From 2005 to 2015, the proportion of people aged 60 or more increased from 9.8% to 14.3%. It is estimated that by 2070 the portion of the elderly will correspond to 35% of the Brazilian population ^1^, which indicates the need for public health to turn to such projections. In general, the changes in physical, physiological, psychological, and social spheres related to aging can be easier if a more-active lifestyle is adopted ^2^. The recommendations of the World Health Organization (WHO) regarding care strategies to respond to the demands of the population aging in the world refer precisely to physical activity ^3^ considering that physical inactivity is responsible for 9% of deaths worldwide ^4^, which means it is the fourth mortality risk factor ^3^.

Older adults should get at least 150 minutes of moderate physical activity per week in series of at least 10 minutes continuously or 75 minutes of vigorous intensity per week in series of at least 10 minutes continuously in any of these four areas: Work, commuting/transport home, and leisure ^3^.However, besides not achieving the recommended values of physical activity, older people tend to spend too much time on tasks that require minimal energy expenditure, such as staying in a sitting position ^5^^,^^6^.

In this context, we have to consider sedentary behavior, which refers to the exposure to activities that require energy expenditure slightly higher than sitting, reclining, or lying during wakefulness. Watching TV, using the computer or mobile phone, working or studying in a table are examples of activities that require low energy expenditure ^7^. Older adults are the age group more exposed to sedentary behavior amounting to 65 to 80% of their waking time ^6^. There is evidence linking this exposure with an increased risk of mortality from all causes, chronic diseases such as diabetes mellitus, cardiovascular disease, and obesity ^8^.

Although they may be analyzed similarly, sedentary behavior and physical inactivity are not the same, as they are constructs with different determinants and physiological health-related responses. Sedentary behavior does not imply the absence of physical activity or non-compliance with physical activity recommendations (<150 min/week) ^9^^,^^10^. In this sense, it is possible to combine the two constructs based on the interaction of both behaviors, i.e., individuals may display enough physical activity up to the minimum of 150 minutes per week and still spend many hours a day in sedentary activities, or spend a few hours dedicated to low-energy expenditure activities similar to rest or accumulated levels of physical activity. The two components may be also negatively affected, which is the least favorable scenario, or they may be both satisfactory, which is the ideal condition for health ^9^. In this sense, these behaviors are not mutually exclusive ^7^ and they are influenced by historical conjunctures, technological apparatus, and modern lifestyle leading to setbacks in habits and routines. Today, much less time and intensity are spent in physical activity while much more time is dedicated to sedentary behavior ^5^^,^^11^.

Sedentary behavior is an emerging topic in public health, but the combined approach with physical activity allowing for deeper scrutiny of their relationship and their implications for health is even more recent ^5^^,^^12^. Furthermore, cross-sectional studies predominate over longitudinal ones ^5^^,^^13^ that enable establishing relations of cause and effect. In this context, our study aimed at verifying the transition and the factors related to the level of physical activity combined with sedentary behavior among community elders followed for 24 months.

## Materials and methods

We conducted an observational, longitudinal, prospective, quantitative study among individuals aged 60 years or over with no cognitive decline residing in the urban area of the municipality of Uberaba, Brazil. The data were collected from January to April, 2014, and from April to July, 2016, in an elderly care home by trained interviewers after informed consent. The initial selection was done with multistage cluster sampling considering a 95% confidence interval, 80% power, 4% error rate for interval estimates, and π=0.5 for the proportions of interest, which resulted in 816 older adults. Details about the sample selection were described in previous publications ^14^. There were 106 losses in the first evaluation due to census tracts with no elderly (n=32), no residence (n=36), an incomplete number of elderly (n=19), and a lack of data on the elderly (n=19).

Of the 710 older adults interviewed in 2014, 374 completed the follow-up in the second evaluation in 2016 after the full interview. The others had cognitive decline (85), refused to participate (42), were not found after three attempts of contact (65), died (39), moved (55), were hospitalized ^10^, and for other reasons, namely addresses not found and missing data ^40^.

We obtained data on gender (male / female), age group (60 to 79 years /80 or over), education (no education / 1-4 years/ 5 or more), marital status (no companion / companion), occupation (yes / no), income (no / up to one minimum wage / 2 or more), housing (alone / with others), health perception (negative / positive), morbidities (no morbidities / 1-4 / 5 or more), hospitalization in the last 12 months (yes / no), and falls in the last 12 months (yes / no). We divided age groups into two: younger old (60 to 79 years) and older old (80 years or over) due to the conceptual relevance of dichotomization, already usual in the literature, the sample size, and the ease for the interpretation of results. Having two heterogeneous and contrasting groups allows for a better understanding of the impact of this variable on the outcome.

Also, we used the Mini-Mental State Examination (MMSE) for the cognitive screening test considering education as the cutoff point ^15^ and the Basic Activities of Daily Living Scale (Katz scale) ^(^^16^, which allowed us to classify the elderly between dependent or independent. We evaluated the Instrumental Activities of Daily Living (IADL) using the Lawton and Brody Scale and we subsequently classified between dependent or independent persons ^17^.

We used the Short Physical Performance Battery (SPPB) to evaluate the physical performance with tests of balance, gait speed, and strength of the lower limbs classifying participants into four categories: Inability or very bad performance, low performance, moderate performance or good performance ^18^.

The level of physical activity was estimated using the International Physical Activity Questionnaire (IPAQ) in its long version ^19^. This instrument addresses issues concerning the minutes spent on physical activities of moderate to vigorous intensity performed during a usual week. The individuals were considered sufficiently active when they spent 150 minutes or more per week on tasks in the four domains of the IPAQ: Work, transportation, housework, and leisure activities. Those accumulating less than 150 minutes were classified as insufficiently active ^20^.

For measuring the sedentary behavior, we used the 5th section of the IPAQ, which determines the time an individual remains seated or awake during a usual day of the week and one during the weekend ^19^. We considered the time spent sitting in different situations (while resting, during meals, reading, watching television, handling electronic devices, and visiting or in other similar contexts) and in many places (home, work, church, offices, community groups, among others). The exposure to sedentary behaviors was calculated based on the weighted average [(week x 5) + (end of week x 2)]/ 7. In the absence of a cutoff point for sedentary behavior in the literature, we used percentile 75 of the time spent seating from the first moment of the evaluation (2014) up to the classification of high sedentary behavior (≥P75) or low one (<P75), a procedure used in other similar studies ^21^^-^^23^; in the present study it corresponded to 420 minutes/day with low sedentary behavior corresponding to 0 to 419 minutes of the time spent seating in a week-day and high to 420 or more minutes of the time spent seating in a week-day.

**Figure 1:**
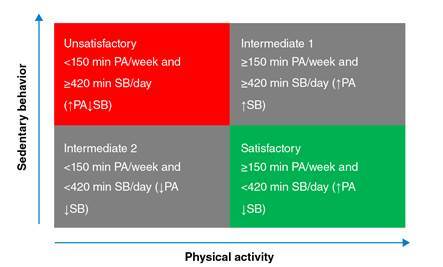
Categorization of the variable level of physical activity combined with sedentary behavior

The evaluation of the physical activity level combined with sedentary behavior (time seated) was based on the cutoff point of 150 minutes per week and in the 75 percentile value, respectively (figure 1) and we established four categories of analysis for each combined variable. Subsequently, we grouped intermediate classes 1 and 2 (with only one of the variables being unfavorable) in three categories: Unsatisfactory, intermediate, and satisfactory.

Data were entered into a Microsoft Office Excel^™^ spreadsheet and later transferred to the Statistical Package for Social Sciences^™^ (SPSS), version 21.0. We conducted the descriptive statistical analysis, of percentages and absolute frequencies, measures of central tendency and variability, and chi- square test to compare proportions. The adjusted analysis was performed by a multinomial logistic regression-non-parametric Wilcoxon test.

The project was approved by the Research Ethics Committee of the Federal University of Triângulo Mineiro, under registration No. 493.211 and 573.833.

## Results

Table 1 compares the categories of the physical activity levels combined with sedentary behavior according to the sociodemographic data of the elderly in 2014 and 2016. It is worth noting that during the two periods evaluated the highest proportion of unsatisfactory and intermediate groups corresponded to the elderly aged 80 years or older while the group of satisfactory level corresponded to the 60 to 79-year-old participants (p=0.002 and p<0.001, respectively). Older adults with no companion prevailed in the unsatisfactory group at baseline in contrast to a higher incidence of those with a partner in intermediate and satisfactory categories (p=0.021). There was a prevalence of elderly who did not have any professional activity in the unsatisfactory and intermediate groups while those with some professional activity prevailed in the satisfactory category during the two periods analyzed (p<0.001).

**Table 1:** Distribution of absolute and relative frequencies of sociodemographic and economic variables of the elderly according to the level of physical activity combined with sedentary behavior at baseline and after 24 months, Uberaba, MG, Brazil, 2014 and 2016

	Physical activity combined with sedentary behavior							
	Unsatisfactory		Intermediate		Satisfactory		p*	p*
Variables	2014 n (%)	2016 n (%)	2014 n (%)	2016 n (%)	2014 n (%)	2016 n (%)		
Gender								
Male	16 (12.6)	10 (7.9)	31 (24.4)	51 (40.2)	80 (63.0)	66 (52.0)	0.496	0.326
Female	24 (9.7)	23 (9.3)	53 (21.5)	80 (32.4)	170 (68.8)	144 (58.3)		
Age group (years)								
60 to 79	28 (8.7)	18 (5.8)	69 (21.4)	100 (32.4)	225 (69.9)	191 (61.8)	0.002	<0.001
80 or more	12 (23.1)	15 (23.1)	15 (28.8)	31 (47.7)	25 (48.1)	19 (29.2)		
Education								
No education	6 (10.7)	6 (10.7)	16 (28.6)	21 (37.5)	34 (60.7)	29 (51.8)	0.194	0.461
1 to 4 years	26 (13.2)	17 (8.6)	37 (18.8)	75 (38.1)	134 (68.0)	105 (53.3)		
5 or more	8 (6.6)	10 (8.3)	31 (25.6)	35 (28.9)	82 (67.8)	76 (62.8)		
Marital status								
No companion	30 (14.8)	21 (10.4)	43 (21.2)	75 (37.1)	130 (64.0)	106 (52.5)	0.021	0.242
Companion	10 (5.8)	12 (7.0)	41 (24.0)	56 (32.6)	120 (70.2)	104 (60.5)		
Professional activity								
Yes	18 (6.7)	18 (6.9)	51 (18.9)	77 (29.5)	201 (74.4)	166 (63.6)	<0.001	<0.001
No	22 (21.2)	15 (13.3)	33 (31.7)	54 (47.8)	49 (47.1)	44 (38.9)		
Income								
No income	-	-	6 (18.2)	12 (34.3)	27 (81.8)	23 (65.7)	0.103	0.283
Up to a salary	25 (14.2)	14 (9.0)	39 (22.2)	59 (37.8)	112 (63.6)	83 (53.2)		
Two or more salaries	15 (9.1)	19 (10.4)	39 (23.6)	60 (32.8)	111 (67.3)	104 (56.8)		
Housing								
Alone	11 (13.6)	9 (11.1)	23 (28.4)	31 (38.3)	47 (58.0)	41 (50.6)	0.163	0.475
Accompanied	29 (9.9)	24 (8.2)	61 (20.8)	100 (34.1)	203 (69.3)	169 (57.7)		

* Chi square test, p<0.05

Table 2 shows the categories of physical activity level combined with sedentary behavior according to the health data of the elderly in 2014 and 2016. The negative perception of health among the elderly of unsatisfactory and intermediate categories at the moment of the final evaluation was evidenced while those classified as satisfactory had positive self-perceived health status (p=0.038). Regarding basic daily life activities, there was a greater proportion of dependent elderly in the unsatisfactory and intermediate groups and independent people in the satisfactory category at baseline (p=0.037). This feature was also observed in instrumental activities of daily living in the two evaluation periods (p<0.001). Regarding the physical performance of the lower limbs, the lowest response (worst performances) was registered among the elderly in the unsatisfactory and intermediate groups as compared with the satisfactory group both in 2014 and 2016 (p<0.001).

**Table 2:** Distribution of absolute and relative frequencies of the health variables of the elderly according to the level of physical activity combined with sedentary behavior at baseline and after 24 months, Uberaba, MG, Brazil, 2014 and 2016

	Physical activity combined with sedentary behavior							
	Unsatisfactory		Intermediate		Satisfactory		p*	p*
Variables	2014 n (%)	2016 n (%)	2014 n (%)	2016 n (%)	2014 n (%)	2016 n (%)	2014	2016
Health perception								
Negative	26 (12.2)	23 (11.3)	51 (23.9)	77 (37.9)	136 (63.8)	103 (50.7)	0.332	0.038
Positive	14 (8.7)	10 (5.8)	33 (20.5)	54 (31.6)	114 (70.8)	107 (62.6)		
Morbidities								
None	2 (11.1)	-	2 (11.1)	2 (25.0)	14 (77.8)	6 (75.0)	0.390	0.508
1 to 4	10 (7.3)	15 (11.1)	32 (23.4)	43 (31.9)	95 (69.3)	77 (57.7)		
5 or more	28 (12.8)	18 (7.8)	50 (22.8)	86 (37.2)	141 (69.4)	127 (55.0)		
Hospitalization								
Yes	11 (17.5)	4 (7.8)	15 (23.8)	21 (41.2)	37 (58.7)	26 (51.0)	0.132	0.612
No	29 (9.3)	29 (9.0)	69 (22.2)	110 (34.1)	213 (68.5)	184 (57.0)		
Falls								
Yes	13 (14.4)	11 (12.1)	27 (30.0)	37 (40.7)	50 (55.6)	43 (47.3)	0.033	0.120
No	27 (9.5)	22 (7.8)	57 (20.1)	94 (33.2)	200 (70.4)	167 (59.0)		
BADL								
Independent	37 (10.1)	30 (8.4)	82 (22.4)	124 (34.6)	247 (67.5)	204 (57.0)	0.037	0.195
Dependent	3 (37.5)	3 (18.8)	2 (25.0)	7 (43.8)	3 (37.5)	6 (37.5)		
IADL								
Independent	4 (2.3)	2 (1.8)	34 (19.9)	26 (23.2)	133 (77.8)	84 (75.0)	<0.001	<0.001
Dependent	36 (17.7)	31 (11.8)	50 (24.6)	105 (40.1)	117 (57.6)	126 (48.1)		
SPPB								
Very bad	13 (56.5)	10 (35.7)	9 (39.1)	13 (46.4)	1 (4.3)	5 (17.9)	<0.001	<0.001
Low	12 (23.5)	7 (11.5)	16 (31.4)	31 (50.8)	23 (45.1)	23 (37.7)		
Moderate	7 (5.0)	14 (9.1)	32 (22.7)	58 (37.7)	102 (72.3)	82 (53.2)		
Good	8 (5.0)	2 (1.5)	27 (17.0)	29 (22.1)	124 (78.0)	100 (76.3)		

BADL: Basic Activities of Daily Living; IADL: Instrumental Activities of Daily Living; SPPB: Short Physical Performance Battery

* Chi square test, p<0.05

There was a decrease in the number of the elderly in the satisfactory (10.6%) and unsatisfactory (1.9%) groups, as well as an increase in the intermediate category (12.5%), with a greater variation and a statistically significant difference (p=0.026) (table 3). In fact, a large percentage of older adults left the satisfactory condition and migrated to the intermediate one (17.6%), which means their health condition deteriorated. In contrast, 5.3% of the aged moved from the unsatisfactory to the intermediate category indicating a positive evolution from the perspective of physical activity and sedentary behavior constructs.

**Table 3:** Evolution of the level of physical activity combined with sedentary behavior among the elderly during follow-up, Uberaba, MG, Brazil, 2014 and 2016

	Evolution of physical activity level combined with sedentary behavior		
	2014 n (%)	2016 n (%)	p*
Satisfactory (PA ↑ ↓ SB)	250 (66.8)	210 (56.2)	
Intermediate (PA ↑ ↑ SB) (PA ↓ ↓ SB)	84 (22.5)	131 (35.0)	0.026
Unsatisfactory (PA ↓ ↑ SB)	40 (10.7)	33 (8.8)	

PA: Physical activity; SB: Sedentary behavior

* p<0.05, Wilcoxon test

Variables with p<0.20 in the crude analysis were eligible for analysis by the multinomial logistic regression model. After adjustment, they kept a greater chance of progression to the unsatisfactory and intermediate categories for those participants with no professional activity compared to those who worked. Not having an occupation increased by 3.03 times the chance of transition to the intermediate group and 5.47 to the unsatisfactory group (p<0.001). A similar behavior in terms of moving to the intermediate and unsatisfactory groups was observed among those with low scores on the assessment of lower limbs physical performance as opposed to those who had high scores (p<0.001). Older adults aged 80 or older were 2.93 times more likely to move to the unsatisfactory group (p=0,031) than the younger elderly. Similarly, those dependent for instrumental activities of daily living were 4.24 times more likely to migrate to the unsatisfactory group (p=0.013) compared to the independent elderly (table 4).

**Table 4:** Multinomial logistic regression for variables associated with physical activity combined with sedentary behavior among the elderly, Uberaba, MG, Brazil, 2014 and 2016

		PA and SB Adjusted Analysis				
Variables	Intermediate			Unsatisfactory		
	OR	CI95%	p*	OR	CI95%	p*
Age group (years)						
60 to 79		1			1	0.031
80 or more	1,433	0.679 to 3.023	0.345	2,93	1.102 to 7.793	
Education						
No education	1,448	0.734 to 2.856	0.286	1,468	0.543 to 3.968	0.450
Education		1			1	
Professional activity						
Yes		1			1	
No	3,036	1.710 to 5.389	<0.001	5,472	2.355 to 12.716	<0.001
Health perception						
Negative	1,127	0.645 to 1.967	0.675	1,129	0.472 to 2.703	0.785
Positive		1			1	
IADL						
Dependent	1,157	0.666 to 2.011	0.604	4,242	1.358 to 13.254	0.013
Independent		1			1	
SPPB (score)	0.789	0.704 to 0.883	<0.001	0.649	0.559 to 0.754	<0.001

IADL: Instrumental Activities of Daily Living; SPPB: Short Physical Performance Battery; OR: Odds ratio; CI: Confidence interval

Reference category: satisfactory

* Chi square test, p<0.05

## Discussion

This survey confirmed the transition among the factors related to the combination of physical activity and sedentary behavior in a sample of community older adults.

The descriptive results showed consistency to detect lower levels of physical activity and longer time of sedentary behavior in agreement with other studies among the oldest seniors ^24^ with no professional activity ^25^, poor health perception, limitations for daily living activities, and low physical performance ^26^.

However, studies were inconclusive regarding gender, education, and income, factors mostly associated with reduced levels of physical activity and extended periods of sedentary behavior ^27^^,^^28^, which alludes to the behavior such as the socio- cultural, historical, and environmental existence of other variables influencing this factors ^25^^,^^29^.

It is noteworthy that, in addition to the methodological heterogeneity applied in the studies, most of them assessed physical activity and sedentary behavior variables independently and not in a combined form thus limiting the scope of possible comparisons with our results and hindering more accurate observations and assertive inferences. In general, an increase in the time of sitting as age advances is to be expected. However, in our case, the short follow-up period to guarantee obtaining data by self-report may have contributed to the decrease of 11.06% in sedentary behavior.

As for the combined evaluation of the constructs, a population-based cross-sectional study conducted among 452 elderly with the same instrument and cutoff point we used (150 min for physical activity and percentile 75 for sedentary behavior) also reported a higher percentage of subjects with satisfactory levels of physical activity and sedentary behavior (n=205; 45.4%) in the combined evaluation. There was a higher percentage for those with low physical activity and sedentary behavior (n=142; 31%) classified in the intermediate 2 group in our study. Thirty-five (7.7%) of the individuals were included in the intermediate 1 group, i.e., that a higher number than in any of the two moments in our study (n=70; 15.5%) had unsatisfactory levels of physical activity and sedentary behavior ^21^.

A similar study in Spain among 433 individuals with 55 years or more collecting data through validated self-report instruments and considering a cutoff point of 3 hours for sedentary behavior found a higher prevalence of inactive subjects with high sedentary behavior (48.9% among men and 42% among women). Besides, more women (27.9%) than men (16.1%) were inactive and had low sedentary behavior, more men (21.0%) than women (15%) were active and had high sedentary behavior and the percentage of men (14.0%) and women (15.0%) who were active and had low sedentary behavior was similar ^12^. These differences in results may be explained by the diversity of instruments and cutoff points adopted.

In our study, although we registered the transition of subjects from the satisfactory to the intermediate group, the percentage of the elderly who remained in the satisfactory category was higher, a finding that may be associated with the short follow-up period, which did not allow large changes related to physical activity and sedentary behavior-related habits.

After adjustment through multinomial logistic regression, there was a greater chance of transition to the intermediate category of the elderly with no professional activity and very poor and low lower limb physical performance. For the unsatisfactory category, besides the absence of professional activity and very poor and low physical performance, the oldest age group and those dependent for performing instrumental activities of daily living were also more likely to migrate.

Other researchers have also reported that the oldest among the elderly spent more time sitting and/or had a lower level of physical activity ^25^^,^^26^^,^^30^^-^^33^. It is assumed that there is a 5% increase per year in the daily time spent in sedentary behavior after the age of 65 years ^34^.

Reductions in physical activity levels and the increase in sedentary behavior are characteristics partially expected among the oldest of the old as a response to biological decline ^35^, especially in socially less-favored regions ^24^. Additionally, psychosocial and environmental factors can elicit a shrinkage in physical activity and sedentary behavior ^28^^,^^30^. For example, approximately 50% of the elderly sedentary behavior is attributed to the leisure domain ^36^ reaching 92.1% among those aged 80 or more ^35^. It may be that in some cultures the increase in the sedentary time dedicated to leisure is understood as a reward for the years of work ^31^. Finnish and Japanese retired elderly aged 65 to 75 have reported more time watching TV than those who still had professional activities ^37^. The modification of physical activity- related habits post-retirement may be linked to the reduction of social relations established in jobs and an increase in the time spent at home ^21^.

It is important to note that retiring does not necessarily have a negative connotation in one^’^s life. As far as physical activity concerns, it is possible to dedicate more time to leisure or even maintain some informal work activity. Besides individual aspects, sociocultural factors may influence the adoption of physical activity patterns after retirement.

Dependence for the performance of instrumental activities in daily living has also been associated with the decrease in the physical activity-sedentary behavior combination in this follow-up. Similarly, the insufficiency of physical activity evaluated separately was associated with dependency on instrumental activities in daily living (PR=1.47) among elderly people living in the community in inner Northeast ^33^. Similarly, higher scores in the Lawton and Brody scale correlated significantly with the two highest tertiles of physical activity in a population of 2,000 Colombian elderly ^38^.

Referring few problems in implementing instrumental activities in daily living was a factor related to a higher chance of achieving the recommended levels of physical activity among those aged 50 or more in five countries with low- and middle-income ^28^, which implies the existence of bidirectional interaction between activity and function in the elderly ^39^. Additionally, in three of these countries (China, México, and South Africa) greater difficulties in instrumental activities in daily living were related to sedentary behavior above 4 hours a day ^28^. This, added to the need for physical activity interventions to minimize the occurrence of disability in daily activities, reiterates the necessity of health education programs for the elderly ^40^.

The decline of the lower limb physical performance was also predictive of the worsening condition of physical activity and sedentary behavior standards. The decline in physical function has been related to aging and subsequent negative effects such as mobility difficulties and disabilities ^38^. Other authors have shown the inverse relationship between sedentary behavior and physical performance ^39^. Consistently, a Swedish prospective study conducted among older adults and elderly identified a declining trend of total physical activity levels and increased sedentary behavior and little physical activity time among those aged 60 or more after 6 years of follow-up ^41^.

A sample of 375 older adults living in Presidente Prudente (São Paulo) that spent a long time in sedentary behavior during leisure had a greater chance of low physical performance (OR=2.35) regardless of the physical activity level ^42^. Along 12.3 years of follow-up, physical function suffered loss among the women aged 50 to 79 who reported a long time spent on sedentary behavior at baseline ^43^. A time of 4 or more daily hours remaining seated had a negative impact on balance, limb strength, upper limb flexibility, gait speed, and resistance among 457 women aged 65 or more ^44^. Similarly, in Portugal, a long time spent in sedentary behavior was negative for muscle strength of upper and lower limbs, agility, dynamic balance, and flexibility of the elderly regardless of the moderate to vigorous physical activity. Parallel to this, despite the time spent in sedentary behavior, high levels of moderate to vigorous physical activity were related to higher resistance and flexibility of the upper limbs ^45^.

The absence of muscle contraction and visible motor stimuli in sitting and lying positions reduced the amount of muscle mass and quality of movement and influenced the physical capacity, and, therefore, the functionality of the elderly ^42^.

Older adults with functional limitations randomized into an intervention group with physical activity and a group of health educational guidance benefited from the increasing gait speed and higher scores on the SPPB after 24 months in the multicenter clinical trial of the Lifestyle Interventions and Independence for Elders Study network (the LIFE Study). There was also a dose-dependent effect as 48 minutes a week of increasing regular physical activity were sufficient to promote physical gains. The incidence of disability was reduced in the highest quartile of physical activity compared to the lowest one (HR=0.23) ^40^.

As in our analysis, a Spanish study categorized and investigated the combination of physical activity and sedentary behavior among 433 older adults and the elderly (55-88 years). Using self-report instruments, the subjects were classified as inactive and with high sedentary behavior (48.9% of men and 42.1% of women), inactive and low sedentary behavior (16.1% of men and 27.9% of women), active and high sedentary behavior (21.0% of men and 15.0% of women), and active and low sedentary behavior (14.0% of men and 15.0% of women) ^12^.

In contradiction with our findings and possibly explained by the methodological differences, the condition corresponding to the unsatisfactory category prevailed while we registered the lowest levels in the satisfactory group. The study, which considered more than 3 hours a day as high sedentary behavior, compared the physical performance of the four groups listed. According to the authors, the worse aerobic endurance and reduced lower limb strength were reported among men of the two inactive groups, regardless of the sedentary behavior. Regarding agility, this was greater in the inactive and low sedentary behavior group. When the six-minute walk and the sit-to-stand tests were performed, there was a significant difference between the inactive and low sedentary behavior groups and the active and low sedentary behavior one, as well as between the inactive and high sedentary behavior groups and the active and low sedentary behavior one ^12^.

Thus, the older adults who devoted more time to physical activity or less time to sedentary behavior had better physical performance ^26^. Although the accumulation of long periods of sedentary behavior and few interruptions are associated with worse health outcomes, there is no consensus on whether physical activity interventions are more effective in reducing the sedentary behavior than those specifically focused on it. Anyway, the social component of the actions must take into account the accessibility of the elderly to the interventions ^13^.

Additionally, the characteristics related to local culture can impact the opportunity and access of specific groups, such as older people, to programs and means of improving the behavioral patterns associated with physical activity ^45^.

Refraining from performing physical activity due to personal constraints, third parties or the environment reinforces the inductive loop of deconditioning and its repercussions on the physical, cognitive, and emotional health ^46^. Therefore, health professionals should be prompted to adopt more positive behaviors regarding physical activity and sedentary behavior.

Considering the potential risks of the combination of low levels of physical activity and high sedentary behavior for health, interventions addressing the improvement of both behaviors should be prioritized ^13^^,^^32^^,^^39^. From a wider perspective, public policies should provide conditions for access and maintenance of healthy habits favoring aging with more quality.

The discrepancies in the results of different studies are partly explained by methodological variations ^8^ in the definitions of the terms, the cutoff points, and the follow-up time, as well as the acquirement methods (objective and subjective measurements) and data analytical management (categorization), the heterogeneity of demographic variables (developed or developing countries), and the health condition of participants, factors that hinder the comparisons and the conclusions on the outcomes.

In the survey we used, given that the follow-up period was 24 months, there was a further evolution of the physical activity and sedentary behavior for the intermediate category than for the unsatisfactory one. Nevertheless, our study allowed for the identification of the relationship between the combined variable and the sociodemographic and clinical characteristics of the elderly population. Our results emphasize the need for practices involving the work of multidisciplinary teams using the elements that make up the every-day reality of the elderly. In this sense, the implications for the practice should consider broader aspects beyond the biological component of physical activity and sedentary behavior.

Some limitations of the study should be taken into account: The high percentage of individuals lost during the follow-up and the difficulty of generalizing the results considering the intrinsic characteristics of the population (demography and cultural conditions).

Our study had a short follow-up period and used questionnaires to survey the variables of physical activity and sedentary behaviors (minimized through proper training of evaluators and the preserved cognition of the elderly verified by using the MMSE for the reminiscent issues). The combined evaluation of the constructs of physical activity and sedentary behavior, as well as the longitudinal design, not widely adopted in studies on the subject yet, stand out as relevant points of this research.

Further studies are required to elucidate the interaction of these two factors in the health of the elderly, a population groups with its peculiarities that, therefore, requires specific interventions.
